# Loss of *NF2* defines a genetic subgroup of non‐*FOS*‐rearranged osteoblastoma

**DOI:** 10.1002/cjp2.172

**Published:** 2020-06-16

**Authors:** Karim H Saba, Louise Cornmark, Jakob Hofvander, Linda Magnusson, Jenny Nilsson, Hilda van den Bos, Diana CJ Spierings, Floris Foijer, Johan Staaf, Otte Brosjö, Vaiyapuri P Sumathi, Suk Wai Lam, Karoly Szuhai, Judith VMG Bovée, Michal Kovac, Daniel Baumhoer, Emelie Styring, Karolin H Nord

**Affiliations:** ^1^ Department of Laboratory Medicine, Division of Clinical Genetics Lund University Lund Sweden; ^2^ European Research Institute for the Biology of Ageing University of Groningen, University Medical Center Groningen Groningen The Netherlands; ^3^ Department of Clinical Sciences, Division of Oncology and Pathology Lund University Lund Sweden; ^4^ Department of Orthopedics Karolinska University Hospital Stockholm Sweden; ^5^ Department of Musculoskeletal Pathology Royal Orthopaedic Hospital Birmingham UK; ^6^ Department of Pathology Leiden University Medical Center Leiden The Netherlands; ^7^ Department of Cell and Chemical Biology Leiden University Medical Center Leiden The Netherlands; ^8^ Bone Tumour Reference Centre at the Institute of Pathology University Hospital and University of Basel Basel Switzerland; ^9^ Department of Orthopedics Lund University, Skåne University Hospital Lund Sweden

**Keywords:** *FOS*, *FOSB*, *NF2*, *WNT5A*, osteoblastoma, osteosarcoma

## Abstract

Osteoblastoma is a locally aggressive tumour of bone. Until recently, its underlying genetic features were largely unknown. During the past two years, reports have demonstrated that acquired structural variations affect the transcription factor *FOS* in a high proportion of cases. These rearrangements modify the terminal exon of the gene and are believed to stabilise both the *FOS* transcript and the encoded protein, resulting in high expression levels. Here, we applied in‐depth genetic analyses to a series of 29 osteoblastomas, including five classified as epithelioid osteoblastoma. We found recurrent homozygous deletions of the *NF2* gene in three of the five epithelioid cases and in one conventional osteoblastoma. These events were mutually exclusive from *FOS* mutations. Structural variations were determined by deep whole genome sequencing and the number of *FOS*‐rearranged cases was less than previously reported (10/23, 43%). One conventional osteoblastoma displayed a novel mechanism of FOS upregulation; bringing the entire *FOS* gene under the control of the *WNT5A* enhancer that is itself activated by FOS. Taken together, we show that *NF2* loss characterises a subgroup of osteoblastomas, distinct from *FOS*‐rearranged cases. Both *NF2* and *FOS* are involved in regulating bone homeostasis, thereby providing a mechanistic link to the excessive bone growth of osteoblastoma.

## Introduction

Osteoblastoma is a bone‐forming tumour that harbours mutations affecting *FOS*, or more rarely its paralogue *FOSB*, in a high proportion of cases [1]. The exact frequency of osteoblastomas with *FOS* or *FOSB* mutations varies depending on the methodology applied. By fluorescence *in situ* hybridisation (FISH) analysis, around 90% of investigated cases have been reported to harbour structural rearrangement of the *FOS* gene [1, 2]. By deep sequencing of a limited number of cases, the *FOS* rearrangements were shown to affect the terminal exon of the gene [1]. The functional outcome is increased FOS expression, likely due to reduced degradation of both *FOS* mRNA and its protein product [3]. By immunohistochemical analysis, 60–80% of osteoblastomas show increased FOS expression, which can be used as a reliable marker in routine clinical diagnostics [2, 4]. Less than 5% of cases display rearrangements of the *FOSB* gene [1, 4]. One case confirmed by deep sequencing analysis harboured a *PPP1R10‐FOSB* fusion gene, in which the coding parts of *FOSB* were placed under the control of the *PPP1R10* promoter. Other FOSB positive cases were found by immunohistochemical or FISH analyses.

Although *FOS* and *FOSB* mutations are frequent findings in osteoblastoma, they do not underlie all cases. We have previously reported complex genome rearrangements including recurrent chromosome 22q12 deletions in osteoblastoma [5]. These complex alterations were found in so‐called epithelioid (previously referred to as aggressive) osteoblastoma. Epithelioid osteoblastoma has the same clinical behaviour as conventional osteoblastoma, i.e. surgery cures most cases, but tumours that are inaccessible or recur can cause considerable morbidity [6]. In addition, osteoblastoma can be diagnostically challenging because its histological features may overlap with those of high‐grade osteosarcoma. Here, we report genetic data from a series of conventional and epithelioid osteoblastomas. Our main finding was complete loss of the *NF2* gene in a subgroup of non‐*FOS‐*rearranged, preferentially epithelioid osteoblastomas.

## Materials and methods

### Tumour material

Material from 29 osteoblastomas was collected from the Skåne University Hospital and the Karolinska Hospital in Sweden, the University Hospital Basel in Switzerland, the Leiden University Medical Center in the Netherlands, and the Royal Orthopaedic Hospital in Birmingham, UK. Five of the tumours were classified as epithelioid osteoblastoma, and 24 as conventional osteoblastoma. Patient age ranged from 2 to 50 years (mean and median ages 23 and 17 years, respectively), and two‐thirds were males (20/29). Detailed patient information can be found in the supplementary material, Table S1. The study was approved by the Institutional Review Boards of the participating centres.

### Genome‐wide copy number and structural analyses of bulk tumour DNA

DNA was extracted from fresh frozen tumour biopsies according to standard procedures [7]. SNP array analysis was performed using the CytoScan HD arrays and Chromosome Analysis Suite v 4.0 (Thermo Fisher Scientific, Waltham, MA, USA), and the Illumina Human Omni‐Quad BeadChips and GenomeStudio software (Illumina, San Diego, CA, USA) [5, 7]. SNP array findings in seven of the cases have previously been published, including two aberrant and five normal profiles (see supplementary material, Table S1) [5]. Whole genome mate pair sequencing was carried out using the Nextera Mate Pair Library Preparation Kit (Illumina) [7]. Sequencing depth was 2.4× on average (mapping coverage 1.7×) and the mean insert size was 3.0 kb, resulting in a median spanning coverage of 45.2× of the human genome (mean 48.1×, range 25.7–100.1×). Sequencing reads were trimmed using NxTrim v 0.4.2 and then aligned against the GRCh37/hg19 build using the Borrows‐Wheeler Aligner v 0.7.15 [8]. To identify structural rearrangements, the sequence data were analysed using Integrative Genomics Viewer, as well as the structural variant callers TIDDIT v 2.7.1 [9] and Delly2 v 0.7.8 [10]. Case 1 and a matched normal control sample were analysed by whole genome paired‐end sequencing using the Complete Genomics platform. Sequencing depth was approximately 100× and 30×, respectively. Case 2 and a matched normal control sample were analysed by whole exome sequencing, as previously described [11]. Copy number plots based on whole genome and whole exome data were created using CNVkit [12].

### Whole genome low‐pass sequencing of individual cells

Whole genome sequencing of cryopreserved primary osteoblastoma cells was performed using a modified single cell whole genome sequencing protocol and 77 base pair single reads were generated using a NextSeq 500 sequencing instrument (Illumina) [13]. Copy number analysis was performed using AneuFinder [14].

### Transcriptome sequencing

RNA was extracted from fresh frozen tumour biopsies according to standard procedures and sequenced using the TruSeq RNA Sample Preparation Kit v2 (Illumina) [7]. Sequencing reads were aligned to the GRCh37/hg19 build using STAR v 2.5.2b [15]. For comparison of relative gene expression levels, gene counts were FPKM (fragments per kilobase per million mapped reads) normalised using Cufflinks with default settings [16], and visualised using the Qlucore Omics Explorer version 3.5 (Qlucore AB, Lund, Sweden). In total, gene expression data from 13 osteoblastomas and, as control, 69 osteosarcomas were available.

### FISH and immunohistochemical analyses

FISH analyses on formalin‐fixed paraffin‐embedded slides and metaphase spreads were performed as described previously [2], using break‐apart probes for *FOS*. Immunohistochemical analysis was performed using a rabbit polyclonal antibody against the N‐terminal region of FOS and a rabbit monoclonal antibody for FOSB [2].

## Results and discussion

**Figure 1:**
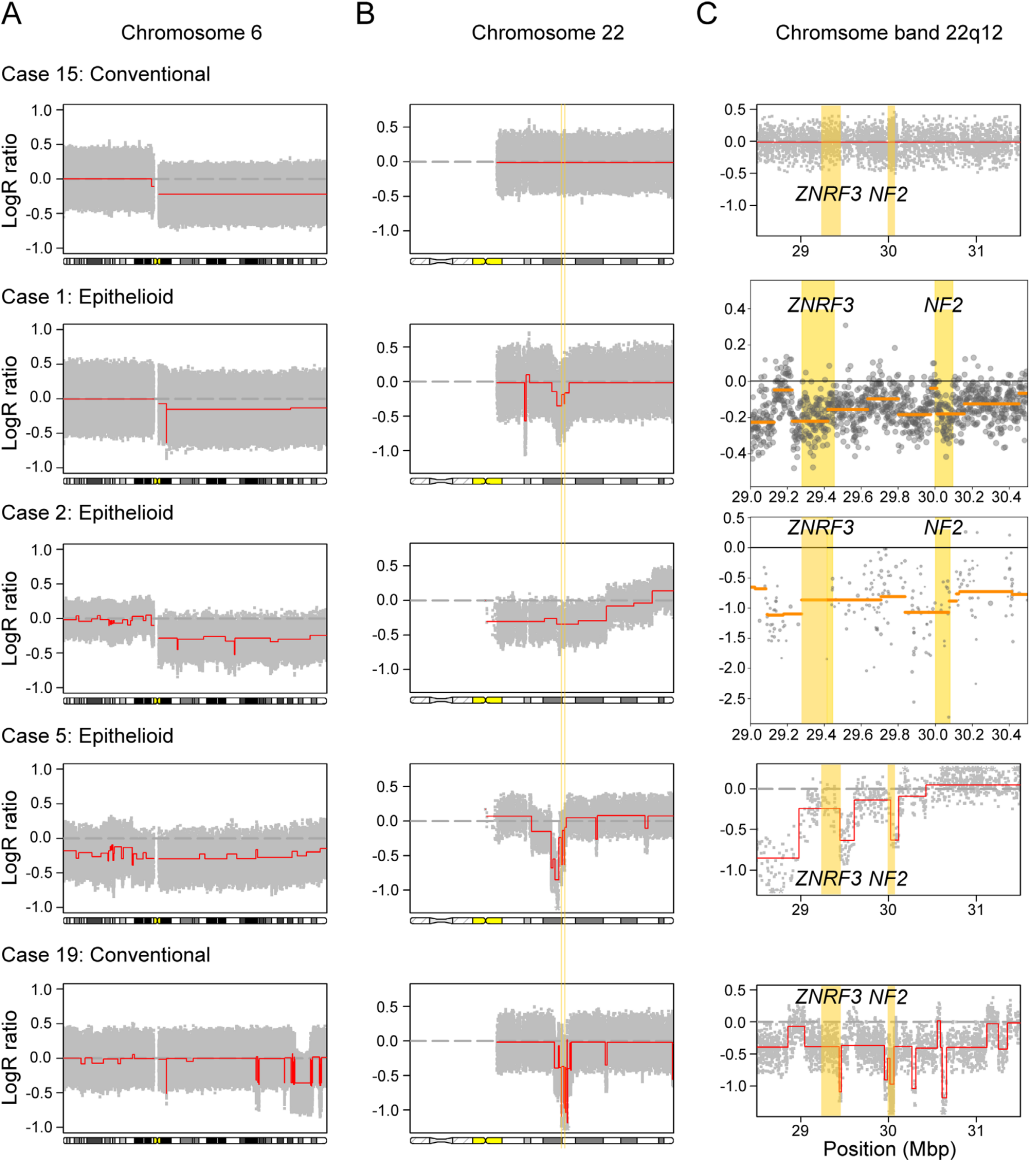
High‐resolution genomic copy number analyses reveal a subgroup of osteoblastoma harbouring recurrent deletions in 6q and 22q12. (A) SNP array analysis detects recurrent deletions in chromosome arm 6q in five osteoblastomas. (B) Four of them harbour concomitant deletions in chromosome arm 22q. (C) Whole genome paired‐end sequencing (Case 1), whole exome sequencing (Case 2), and SNP array analysis (Cases 5 and 19) show that these deletions cluster to the *ZNRF3* and *NF2* genes in 22q12. The latter gene is affected by intragenic homozygous deletions in all four cases. Case 15 did not harbour any deletions affecting chromosome 22. Yellow lines mark the positions of the *ZNRF3* and *NF2* genes.

**Figure 2:**
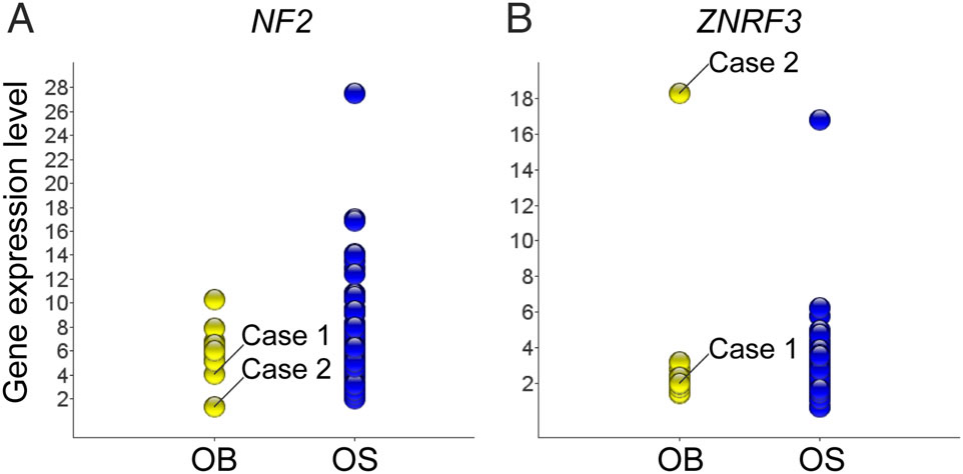
Transcriptome analysis consolidates *NF2* as the most likely target for 22q12 deletions in osteoblastoma. (A) Cases 1 and 2 harbour intragenic homozygous *NF2* deletions and concomitant low *NF2* expression levels, compared with osteoblastomas without detected *NF2* deletion (*n* = 11) and osteosarcomas (*n* = 69). (B) Case 1 harbours an intragenic homozygous deletion and concomitant low expression level of the *ZNRF3* gene. Case 2 harbours a hemizygous loss and relative high expression level of *ZNRF3*. OB, osteoblastoma; OS, osteosarcoma.

**Figure 3:**
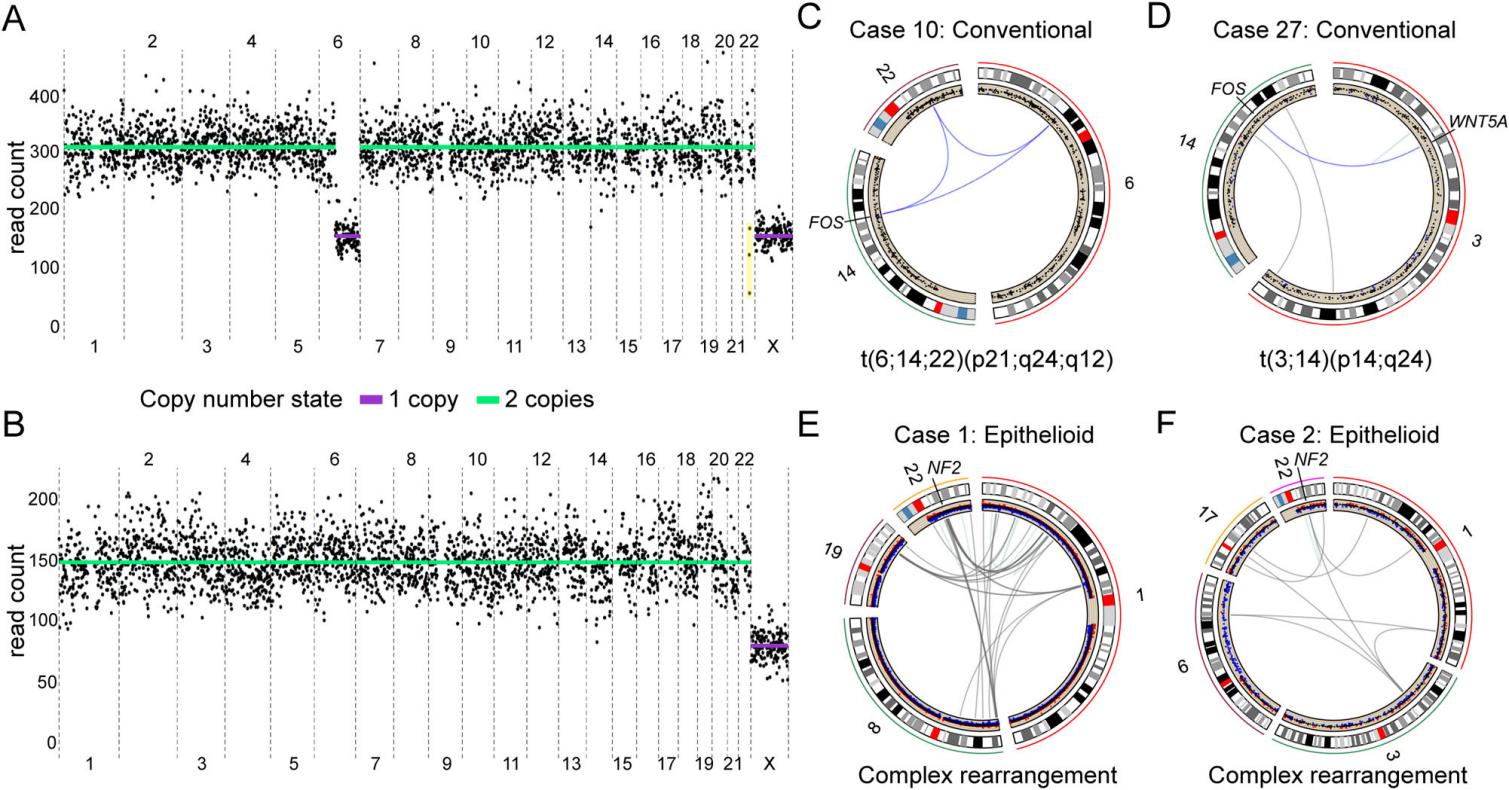
*NF2* deletions and *FOS* structural variations are mutually exclusive in osteoblastoma. (A) Whole genome sequencing of an individual cell from Case 1 shows hemizygous loss of chromosome arm 6q. The patient is male, and a single copy of the X chromosome is detected (the Y chromosome is not shown). The low number of reads mapping to the three copy number bins highlighted in yellow suggest a combination of hemi‐ and homozygous losses across this region (27.7–31.1 Mb, according to genome assembly GRCh37/hg19). (B) A representative cell with no acquired copy number alterations, presumably a non‐neoplastic cell.(C) A balanced three‐way translocation results in structural rearrangement of the 3′ part of the *FOS* gene in Case 10. (D) A balanced two‐way translocation juxtaposes the complete coding region of *FOS* and the enhancer region of *WNT5A* in Case 27. (E,F) Complex structural variations affecting 22q12 and many other chromosomal regions result in genomic copy number imbalances, including intragenic homozygous losses of *NF2* in Cases 1 and 2.

Genomic copy number status was determined in all 24 conventional and five epithelioid osteoblastomas using SNP array analysis. Out of the 29 cases, seven displayed acquired DNA copy number alterations (see supplementary material, Table S2). Five cases harboured hemizygous deletions of either whole or parts of chromosome arm 6q (Figure 1A), of which four also had hemi‐ and homozygous deletions in chromosome arm 22q (Figure 1B). The latter clustered to a minimal deleted region in chromosome band 22q12 (Figure 1C). Combined with copy number and structural variant information from whole exome and whole genome sequencing analyses, we could confirm that the *NF2* gene in 22q12 was affected by intragenic homozygous deletions in all four cases (Figure 1C and see supplementary material, Tables [Link], [Link]). In line with this, the relative expression level of *NF2* was low in affected cases compared with other osteoblastomas and osteosarcomas (Figure 2A). Three out of the four cases with homozygous *NF2* deletions showed intragenic homozygous deletions affecting the *ZNRF3* gene. However, in the remaining *NF2*‐deleted case (Case 2), we detected neither homozygous loss nor low relative expression level of *ZNRF3*, arguing against *ZNRF3* as a target for the deletions (Figures 1C and 2B). The number of ascertained *NF2*‐deleted osteoblastomas is still too low to make any definite correlations with clinical features. However, we noted that three out of four cases with intragenic homozygous *NF2* loss were classified as epithelioid osteoblastoma (see supplementary material, Table S1). We found no acquired genetic alterations in the remaining two epithelioid osteoblastomas. In one of the epithelioid osteoblastomas with 6q and *NF2* losses, we subjected individual cryopreserved cells to whole genome sequencing. Out of 178 individual cells sequenced, 15 (8%) showed acquired copy number variations in agreement with those detected in bulk tumour DNA, i.e. loss of 6q and regions harbouring *NF2* in 22q12 (Figure 3A,B and see supplementary material, Figure S1). Presumably, these cells represent the neoplastic clone and the remaining cells constitute admixed normal cells.

Out of the 23 osteoblastomas for which we could evaluate structural chromosome alterations by whole genome sequencing, *FOS* rearrangements were found in 10 (43%), and *FOSB* rearrangement was not detected (see supplementary material, Tables S1 and S5). This proportion of *FOS* and *FOSB* rearranged cases is lower than what has been found by FISH analyses [1, 2]. Our whole genome sequencing analysis of bulk tumour DNA, with a median spanning coverage of 45× of the human genome, showed only few sequencing reads that supported the *FOS* rearrangements even in positive cases. Thus, deep sequencing analyses of DNA from bulk tumour tissue and individual cells suggest that there is a high proportion of normal cells admixed with the neoplastic clone, and this may obscure the detection of acquired genetic alterations in osteoblastoma.

The *FOS* rearrangements detected here were generated by balanced two‐ or three‐way translocations (Figure 3C,D and see supplementary material, Tables S1 and S5). Few or no other genomic alterations were detected in these cases. Two of our *FOS* rearranged cases are particularly noteworthy. In Case 20, we detected a *FOS‐KIAA1199* fusion (see supplementary material, Table S5), very similar to the *FOS‐KIAA1199* fusion previously reported in osteoblastoma [1]. The competitive advantage, if any, of this particular rearrangement is unknown. In Case 27, we detected a t(3;14)(p14;q24) that did not affect the terminal exon of *FOS*. Instead, the breakpoint in 14q24 was located in one of the *FOS* promoter/enhancer regions, 23 kb upstream of the gene. The translocation placed the complete coding region of the *FOS* gene under the enhancer region of *WNT5A* (Figure 3D and see supplementary material, Table S5)*. WNT5A* is a WNT ligand involved in bone metabolism and is paradoxically induced by FOS [17]. Upregulation of FOS in this case was confirmed by immunohistochemistry (see supplementary material, Table S1). In the present series of osteoblastomas, *FOS* rearrangement was mutually exclusive from *NF2* deletion (see supplementary material, Table S1). While the *FOS*‐rearranged cases displayed balanced chromosome alterations, *NF2‐*deleted cases were characterised by unbalanced, complex rearrangements that affected chromosome band 22q12 and several other chromosomal regions (Figure 3E,F).

We have previously shown recurrent deletions in 22q12 in osteoblastoma, affecting genes that are linked to WNT signalling and bone homeostasis [5]. In the present study, we analysed more cases with a higher resolution. This enabled us to pinpoint the *NF2* gene as the most likely target for the 22q12 deletions in osteoblastoma. In line with this, loss of *NF2* disrupts the Hippo signalling pathway, a key component in osteoclast formation and bone homeostasis [18], and *Nf2‐*deficient mice show increased bone volume [19]. This does not rule out the possibility that additional genes in 22q12, such as *MN1*, *ZNRF3* and/or *KREMEN1*, play a role in osteoblastoma development. However, in support for *NF2*, there is cross talk between NF2 and FOS signalling pathways. More specifically, loss of NF2 will lead to decreased activation of the Hippo pathway, which normally inhibits the activity of the transcriptional co‐activators YAP and TAZ, allowing them to have a longer‐lasting effect in the nucleus [18]. YAP and TAZ also cross activate the AP‐1 transcription factor complex, of which FOS is a main component. YAP/TAZ and AP‐1 can synergistically activate downstream target genes, and a prolonged effect of YAP and TAZ due to loss of NF2 may thereby lead to increased FOS activity and a continued deregulation of signalling pathways.

In summary, we have found mutually exclusive *FOS* rearrangements and intragenic homozygous *NF2* deletions in osteoblastoma. The latter were associated with additional genomic losses, complex structural variations, and clearly clustered to the epithelioid subtype of osteoblastoma. Hitherto, defects in genes that regulate bone homeostasis are common to osteoblastomas.

## Author contributions statement

KHS, ES and KHN conceived and designed the study. KHS, LC, JH, LM, JN, HvdB, DCJS, FF, JS, SWL, KS, JVMGB, MK, DB, ES and KHN generated, analysed or interpreted data. OB, VPS, KS, JVMGB, DB and ES provided tumour material and clinical information. KHS and KHN wrote the manuscript with contributions from all other authors.

## Supporting information


**Figure S1.** Whole genome sequencing of cryopreserved primary osteoblastoma cells from case 1

**Table S1.** Clinical and genetic features of osteoblastoma

**Table S2.** Genomic copy number alterations detected by SNP array analysis

**Table S3.** Genomic copy number state of genes in 22q12

**Table S4.** Structural chromosome alterations affecting the *NF2* gene detected by whole genome paired‐end sequencing and whole genome mate pair sequencing

**Table S5.** Structural chromosome alterations affecting the *FOS* gene detected by whole genome DNA mate pair sequencing
