# Supplementary figures and images for: Loss of NF2 defines a genetic subgroup of non‐FOS‐rearranged osteoblastoma
Source: J Pathol Clin Res. 2020 Jun 16;6(4):231–7. doi: 10.1002/cjp2.172 (PMC7578308; doi:10.1002/cjp2.172)

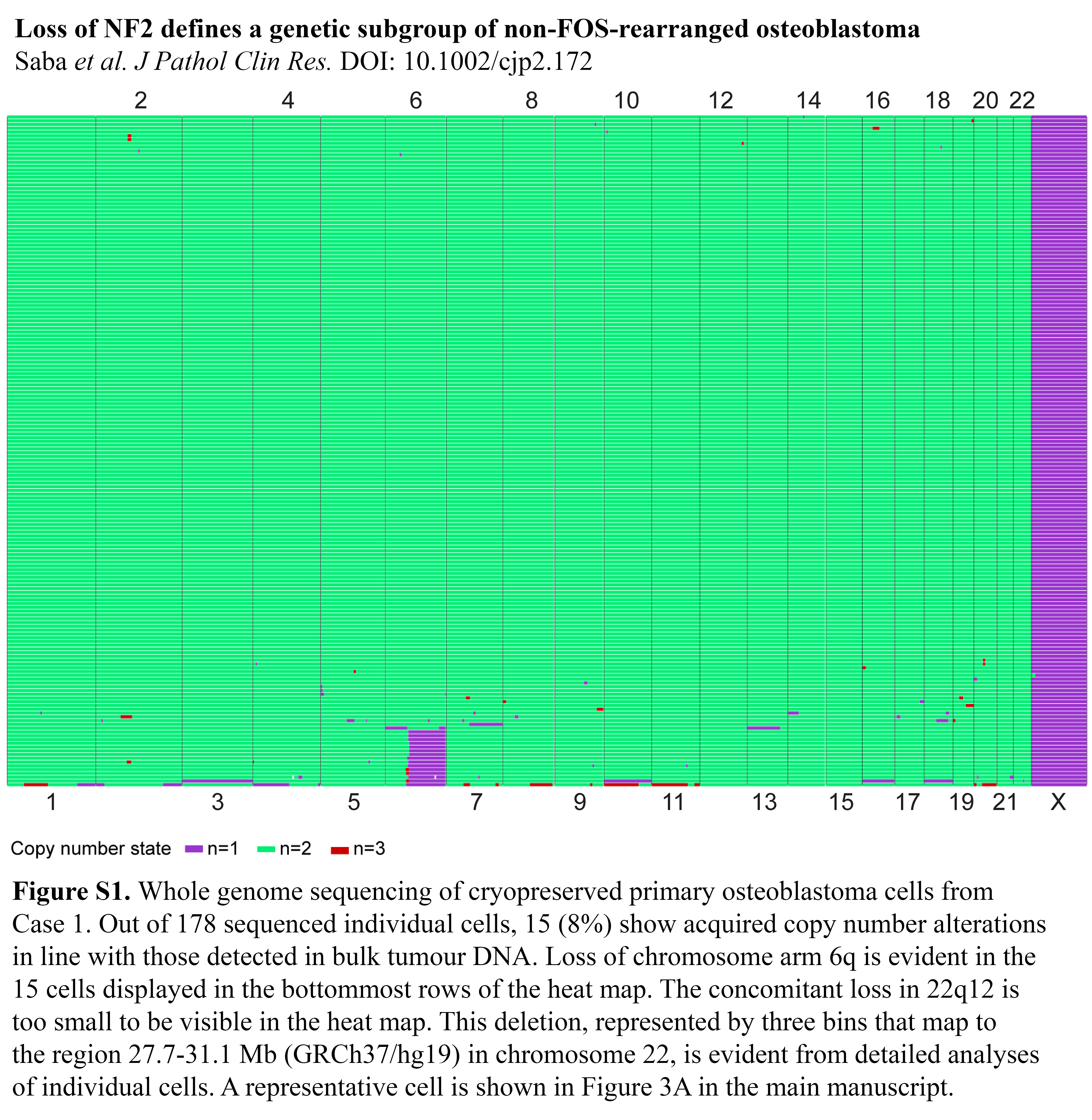

Supplement: Supplementary file 1 — Figure S1. Whole genome sequencing of cryopreserved primary osteoblastoma cells from case 1 [file CJP2-6-231-s001.tif]
